# Use of Spectroscopic, Zeta Potential and Molecular Dynamic Techniques to Study the Interaction between Human Holo-Transferrin and Two Antagonist Drugs: Comparison of Binary and Ternary Systems

**DOI:** 10.3390/molecules17033114

**Published:** 2012-03-12

**Authors:** Mona Kabiri, Zeinab Amiri-Tehranizadeh, Ali Baratian, Mohammad Reza Saberi, Jamshidkhan Chamani

**Affiliations:** 1 Department of Biology, Faculty of Sciences, Mashhad Branch, Islamic Azad University, Mashhad 9175687119, Iran; Email: mona.kabiri@gmail.com; 2 Medical Chemistry Department, School of Pharmacy, Mashhad University of Medical Science, Mashhad 9175687119, Iran; Email: zeinab.amiri86@gmail.com (Z.A.-T.); baratian841@mums.ac.ir (A.B.); saberimr@mums.ac.ir (M.R.S.)

**Keywords:** human holo-transferrin, ropinirole hydrochloride, spectroscopic techniques, zeta potential, molecular modeling

## Abstract

For the first time, the binding of ropinirole hydrochloride (ROP) and aspirin (ASA) to human holo-transferrin (hTf) has been investigated by spectroscopic approaches (fluorescence quenching, synchronous fluorescence, time-resolved fluorescence, three-dimensional fluorescence, UV-vis absorption, circular dichroism, resonance light scattering), as well as zeta potential and molecular modeling techniques, under simulated physiological conditions. Fluorescence analysis was used to estimate the effect of the ROP and ASA drugs on the fluorescence of hTf as well as to define the binding and quenching properties of binary and ternary complexes. The synchronized fluorescence and three-dimensional fluorescence spectra demonstrated some micro-environmental and conformational changes around the Trp and Tyr residues with a faint red shift. Thermodynamic analysis displayed the van der Waals forces and hydrogen bonds interactions are the major acting forces in stabilizing the complexes. Steady-state and time-resolved fluorescence data revealed that the fluorescence quenching of complexes are static mechanism. The effect of the drugs aggregating on the hTf resulted in an enhancement of the resonance light scattering (RLS) intensity. The average binding distance between were computed according to the forster non-radiation energy transfer theory. The circular dichroism (CD) spectral examinations indicated that the binding of the drugs induced a conformational change of hTf. Measurements of the zeta potential indicated that the combination of electrostatic and hydrophobic interactions between ROP, ASA and hTf formed micelle-like clusters. The molecular modeling confirmed the experimental results. This study is expected to provide important insight into the interaction of hTf with ROP and ASA to use in various toxicological and therapeutic processes.

## 1. Introduction

Human holo transferrin (hTf) is a member of the transferrin family that function as iron-binding and iron transport proteins. hTf consists of a single-chain glycoprotein with 679 amino acid residues and has a molecular weight of ~79 kD. The concentration of hTf is approximately 35 µM (2.5 mg/mL). Moreover, hTf has been detected in various body fluids including plasma, bile, amniotic, cerebrospinal and lymph fluids, as well as in breast milk [1,2,3,4]. The hTf folds to form the homologous N-lobe (336 amino acids) and C-lobe (343 amino acids). Each lobe is divided into two sub-domains, which form a deep cleft containing a high-affinity binding site for single ferric iron (Fe^3+^). The saturation of iron in holo-transferrin is only 30% and the vacant sites can bind to numerous other metal ions, leading to uptake by the transferrin receptor (TfR) through endocytosis. The differic transferrin (holo-Tf) is reported to have a higher affinity for TfR than the monoferric and iron-free form (Apo-Tf) [5,6]. Iron in the ferric form is bound to hTf in plasma at neutral pH (pH = 7.4). It is subsequently transported into the cells and released in the acidic environment (pH = 5.5) of the endosome. When iron is released from the N-lobe of hTf, the two domains rotate 63° around a central hinge leading to an open conformation (apo-Tf) [1,2,3,7].

The C-lobe must be able to open in a similar way to the N-lobe, although it binds iron more strongly and releases it more slowly. Moreover, the C-lobe has appeared to be conformationally less flexible. Holo-transferrin has been suggested as a potential drug carrier and delivery system to allow specific targeting to, for instance, cancer cells, since the TfR is over-expressed in a broad range of cancers [8,9]. hTf has been implicated in the transport of drug molecules [10,11], which may help understanding the interaction of drugs with protein molecules in the blood [12,13].

Ropinirole hydrochloride [4-[2-(dipropylamino)ethyl]-1,3-dihydro-2H-indol-2-one monohydro-chloride, ROP, brand-name REQUIP, Figure 1A, is a second-generation non-ergolin dopamine agonist (DA) that selectively activates postsynaptic dopamine receptors. ROP binds with higher affinity to D_3_ than to D_2_ or D_4_ receptor sub-types (D_3_ > D_2_ > D_4_). The molecular weight of ROP is 296.84 Da and it presents a solubility in water of 133 mg/mL [14,15]. ROP is approved for treatment of Restless Leg Syndrome and Parkinson’s disease and its use has been associated with a lower risk of dyskinesias and valvular regurgitation. Recent clinical evidence has shown that the ROP augments the antidepressant effects of many standard drugs such as tricyclic antidepressants (TCA) or selective serotonin reuptake inhibitors (SSRI). Besides, ROP has been reported to possess an anxiolytic and antidepressant profile in various animal paradigms in mice, rats and common marmosets [16,17].

**Figure 1:**
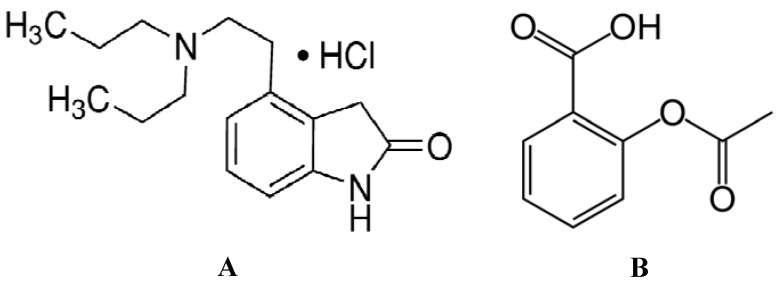
(**A**) The chemical structure of ropinirole hydrochloride (ROP); (**B**) The chemical structure of aspirin (ASA).

Acetylsalicylic acid (ASA) or 2-acetoxy benzoic acid, commonly known as aspirin Figure 1B, is widely applied in a variety of medical areas. ASA is a non-steroidal anti-inflammatory drug (NSAID) that has been used for primary and secondary prevention of artherosclerotic diseases for over two decades. It decreases the onset of cardiovascular disease by 20–25% [18,19]. ASA demonstrates anti-coagulating effects, as well as some respiratory, depressive, antithrombotic, tirheumatic, antipyretic, analgesic and anxiety effects, and prevents strokes, heart attacks and myocardial infarction [20]. ASA may reduce the risk of some types of cancer. Nevertheless, epidemiological evidence also suggests that regular use of ASA and other NSAIDs for an extended period of time increases the risk of pancreatic and kidney cancers [21]. 

This study concerns the characterization by spectroscopic techniques, zeta potential measurements and molecular modeling of the binding of ROP and ASA to hTf under physiological conditions (pH = 7.4). The biological significance of this work is evident since hTf serves as a carrier molecule for multiple drugs. Moreover, the study is also believed to provide important information to clinical research and the theoretical basis for the design of new drugs.

## 2. Results and Discussion

### 2.1. Analysis of the Fluorescence Spectra

Fluorescence quenching is the decrease of the quantum yield of fluorescence from a fluorophore induced by a variety of molecular interactions with a quencher molecule, including excited-state reactions, molecular rearrangements, energy transfer, ground-state complex formation and collisional quenching processes. The fluorescence of hTf comes from the Trp, Tyr and Phe residues. As a matter of fact, the intrinsic fluorescence of hTf is almost exclusively contributed by Trp alone, since Phe has a very low quantum yield and the fluorescence of Tyr is practically totally quenched when ionized or close to an amino group, a carboxyl group, or a Trp. This viewpoint was backed by the experimental observation of Sulkowska *et al*. [22], when a small molecule was bound to hTf, changes of the intrinsic fluorescence intensity of hTf were induced by the micro-environment of the Trp and Tyr residues. The participation of Trp and Tyr groups was assessed through different excitation wavelengths. At a wavelength of 280 nm, both the Trp and Tyr residues in hTf became excited, whereas at 295 nm, this was the case only for the Trp residues. 

Fluorescence spectroscopy is an extremely sensitive technique that has provided a wealth of insight into biological processes [23]. By studying this quenching processes, one can obtain information about the binding of ROP and ASA to hTf, such as the binding mechanism, binding constant and binding site. An intrinsic fluorescence experiment was thus performed to evaluate the interaction of ROP and ASA with hTf. Figure 1A,B displays the fluorescence quenching of hTf induced by ROP and ASA (a single drug), excited at 280 nm. As can be seen, the fluorescence intensity of hTf gradually decreased upon increasing the concentration of ROP or ASA, indicating that the two drugs became bound to hTf. On the other hand, the emission spectra for binary systems portrayed an isobestic point, pointing at the existence of bound and free ROP and ASA in equilibrium. The quenching took place when the quencher was sufficiently close to Trp or/and Tyr residues [24].

**Figure 1:**
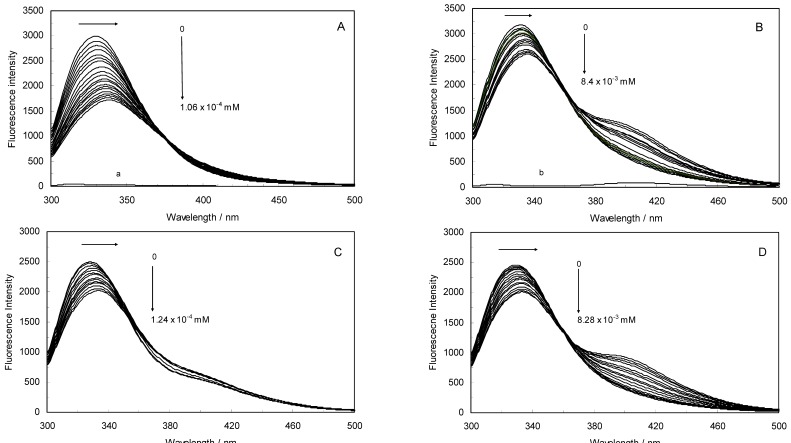
(**A**) Fluorescence emission spectra of the hTf-ROP system. [hTf] = 3.8 × 10^−3^ mM and [ROP] was increased from 0 to 1.06 × 10^−4^ mM; (**B**) Fluorescence emission spectra of the hTf-ASA system. [hTf] = 3.8 × 10^−3^ mM and [ASA] was increased from 0 to 8.4 × 10^−3^ mM; (**C**) Fluorescence emission spectra of the (hTf-ASA)-ROP system. [hTf] = 3.8 × 10^−3^ mM and [ROP] was increased from 0 to 1.24 × 10^−4^ mM, [ASA] = 4.62 × 10^−3^ mM; (**D**) Fluorescence emission spectra of the (hTf-ROP)-ASA system. [hTf] = 3.8 × 10^−3^ mM and [ASA] was increased from 0 to 8.28 × 10^−3^ mM, [ROP] = 1.33 × 10^−5^ mM. The a and b represent the curves of ROP and ASA, respectively. All experiments were performed under identical conditions (T = 298 K; pH 7.4, λ_ex_ = 280 nm). The horizontal arrow shows the red shift.

Figure 1C,D illustrate the fluorescence intensity of hTf by ROP and ASA (ternary systems), excited at 280 nm. The fluorescence of hTf was quenched upon interaction with ROP and ASA, and during the interactions, the isobestic point was observed. Under the experimental conditions, the ROP presented no emission spectra within the 300–500 nm and the emission from ASA was registered in the emission ranges 300–320 nm and 380–500 nm. The shift in maximum wavelength towards longer wavelengths (red shift) for the binary and ternary systems was believed to be due to the conformational changes induced by the interactions, leading to a further exposure of Trp and Tyr residues to a polar solvent and an increased polarity of the fluorophores [25,26].

The overlapping of the quenching curves may indicate that different binding sites of ROP and ASA existed in sub-domains of hTf. In order to confirm the quenching mechanism, the fluorescence quenching data was analyzed by the Stern-Volmer equation:


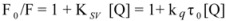
(1)

where F_0_ and F denote the steady-state fluorescence intensities in the absence and presence of a quencher, respectively; K_sv_ is the Stern-Volmer quenching constant; [Q] is the concentration of quencher; k_q_ is the quenching rate constant; and τ_0_ is the average lifetime of the bio-molecule without quencher (*i.e.*, 10^−8^ s) [27,28]. Hence, Equation (1) was applied to determine K_sv_ by linear regression of a plot of F_0_/F against [Q]. The K_sv_ values for binary and ternary systems at different temperatures (298, 308 and 318 K) were calculated and are listed in Table 1. 

**Table 1:** Thermodynamic parameters and K_sv_ values for the drugs when bound to hTf in binary and ternary systems at various temperatures (pH 7.4).

System	T/K	K_SV1_/M^−1^	K_SV2_/M^−1^	ΔG°/kJ mol^−1^	ΔH°/kJ mol^−1^	ΔS°/J mol^−1^	R
		λ *_ex_* = 280 nm	λ *_ex_* = 280 nm				
hTf-ROP	298	(1.16 ± 0.02) × 10^7^	(5.29 ± 0.02) × 10^6^	−36.93 ± 0.32			0.9982
	308	(1.03 ± 0.02) × 10^7^	(5.05 ± 0.02) × 10^6^	−35.71 ± 0.26	−79.17 ± 0.29	−145.25 ± 0.31	0.9983
	318	(9.83 ± 0.02) × 10^6^	(4.92 ± 0.02) × 10^5^	−35.01 ± 0.29			0.9981
(hTf-ASA)ROP	298	(2.34 ± 0.03) × 10^5^	------	−36.17 ± 0.21			0.9976
	308	(2.13 ± 0.03) × 10^5^	------	−35.83 ± 0.31	−71.54 ± 0.32	−120.22 ± 0.29	0.9983
	318	(2.02 ± 0.03) × 10^5^	------	−35.14 ± 0.27			0.9981
hTf-ASA	298	(2.94 ± 0.02) × 10^4^	------	−37.14 ± 0.27			0.9978
	308	(2.47 ± 0.02) × 10^4^	------	−36.21 ± 0.27	−69.32 ± 0.14	−110.36 ± 0.31	0.9980
	318	(2.15 ± 0.02) × 10^4^	------	−35.41 ± 0.31			0.9977
(hTf-ROP)ASA	298	(3.35 ± 0.03) × 10^4^	------	−36.81 ± 0.33			0.9985
	308	(3.02 ± 0.03) × 10^4^	------	−36.04 ± 0.27	−81.21 ± 0.30	−151.76 ± 0.28	0.9991
	318	(2.77 ± 0.03) × 10^4^	------	−31.11 ± 0.30			0.9983

The results demonstrated that the values of Stern-Volmer quenching constants, K_sv_, decreased with rising temperatures, which indicated that the possible quenching mechanism of fluorescence of hTf by ROP and ASA, were of the static quenching type. Furthermore, the maximum diffusion collision quenching rate constant, k_q_, of various quenchers with the biopolymer is 2.0 × 10^10^ L mol^−1^ s^−1^, which revealed that the probable quenching mechanism of binary and ternary systems were initiated by complexes formation rather than by dynamic collision. Since static quenching is due to ground-state complexes formation between the fluorophore and quenchers [23,24,25,29].

**Figure 2:**
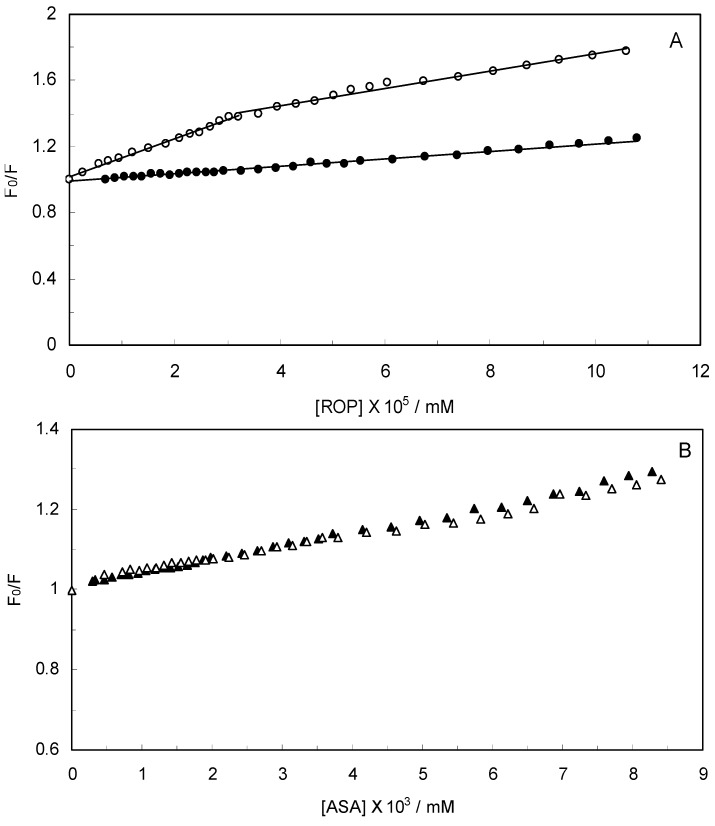
(**A**) Stern-Volmer plots for the binding of ROP to hTf in the absence and presence of ASA. [ASA] = 4.62 × 10^−3^ mM, binary system (○); ternary system (●); (**B**) Stern-Volmer curves for the binding of ASA to hTf in the absence and presence of ROP. [ROP] = 1.33 × 10^−5^ mM, binary system (∆); ternary system (▲).

The Stern-Volmer quenching constant increased with an increasing affinity binding of hTf, ROP and ASA. Figure 2 displays the Stern-Volmer plots of binary (hTf-ROP or ASA) and ternary [(hTf-ROP)-ASA and (hTf-ASA)-ROP] systems excited at 280 nm. The Stern-Volmer dependencies of hTf-ROP (Figure 2A) demonstrated a deviation from the straight line when the concentration of ROP was higher than 3.22 × 10^−5^ mM. Therefore, the hTf could be quenched by two binding sites with different interaction behavior. The quenching was not purely collisional but may be due to the formation of either the ground-static complex or the static quenching process. Alternatively, there might be more than one independent binding site on the hTf for ROP and they are not all accessible to the hTf-ROP complex. The Stern-Volmer linear curves for (hTf-ASA)-ROP, (hTf-ASA) and (hTf-ROP)-ASA (see Figure 2B) revealed that static quenching occurred when these drugs approached the environment of the hTf fluorophores.

### 2.2. Binding Parameters

When small molecules bind independently to a set of equivalent sites on a macromolecule, the binding constant (K_b_) and the number of binding sites (n) can be determined by the following equation: 


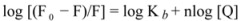
(2)

where F_0_ and F are the fluorescence intensity of the protein in the absence and presence of a quencher, and [Q] is the quencher concentration [30]. The values of K_b_ and n for binary and ternary systems were calculated and are presented in Table 2.

**Table 2:** The binding constants (K*_b_*) and the number of binding sites (n), for the binary and ternary systems at pH = 7.4 and T = 298 K.

System	K *_b_* ( L mol^−1^)	n	R	K *_b_* (L mol^−1^)	n	R
	λ *_ex_* = 280 nm	λ *_ex_* = 280 nm		λ *_ex_* = 295 nm	λ *_ex_* = 295 nm	
hTf-ROP	K *_b_*_1_ = (1.69 ± 0.03) × 10^6^	N_1_ = 0.82	0.997	(3.75 ± 0.03) × 10^8^	1.52	0.998
	K *_b_*_2_ = (1.86 ± 0.03) × 10^5^	N_2_ = 0.59	0.998			
hTf-ASA	(7.83 ± 0.02) × 10^3^	0.74	0.999	(5.51 ± 0.02) × 10^3^	0.78	0.998
(hTf-ASA)-ROP	(8.42 ± 0.02) × 10^5^	1.14	0.998	(5.44 ± 0.02) × 10^6^	1.20	0.996
(hTf-ROP)-ASA	(2.06 ± 0.02) × 10^4^	0.89	0.997	(3.58 ± 0.02) × 10^4^	1.19	0.995

According to Equation (2), the Hill plot of hTf-ROP can be fitted with two lines, which would indicate the presence of two binding sites with different K_b _values. Each linear function represents the ROP interaction to hTf with special behavior, whereas one saturable binding site was found for hTf-ASA in the absence and presence of ROP. The strong binding of ROP to hTf took place or that the transfer of energy from excited fluorophores to ROP molecule facilitated. This suggests that the hTf fluorescence was significantly more quenched by ROP. The decrease in binding constant revealed the availability of higher concentrations of free drugs in the plasma.

### 2.3. Analysis of Second Derivative Fluorescence Spectra

**Figure 3:**
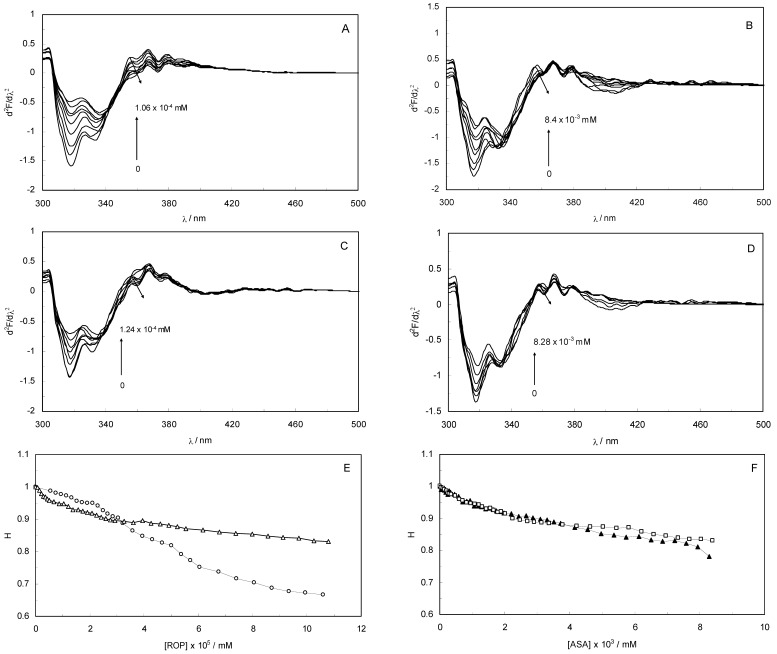
(**A**) The second derivative fluorescence spectra of hTf in the presence of ROP; (**B**) The second derivative fluorescence spectra of hTf in the presence of ASA; (**C**) The second derivative fluorescence spectra for the (hTf-ASA)-ROP system; (**D**) The second derivative fluorescence spectra for the (hTf-ROP)-ASA system; (**E**) A plot of the H versus [ROP] for hTf-ROP in the absence and presence ASA. Binary system (○); ternary system (∆). [ASA] = 4.62 × 10^−3^ mM for the ternary system; (**F**) A plot of the H versus [ASA] for hTf-ASA in the absence and presence ROP. Binary system (□); ternary system (▲). [ROP] = 1.33 × 10^−5^ mM for the ternary system.

Second derivative fluorescence spectroscopy is a sensitive and reliable technique for monitoring and characterizing the transitions that take place in the Trp micro-environment of proteins. Figure 3A–D display the second derivative fluorescence spectra for binary and ternary systems, featuring two negative bands at 319 nm and 334 nm. The origin of these bands was presumably the transition of the electrons back to the different vibration levels of the ground state. When the concentrations of ROP and ASA in the binary and ternary systems were increased, the intensity of the shortest wavelength band was altered. The increase in the intensity of the shortest wavelength band was observed upon increasing the concentration of ROP and ASA, signifying that the binding of ROP and ASA to hTf became affected in addition to conformational changes in hTf and the micro-environment of the Trp residues. As can be seen in Figure 3 the negative band at 319 nm for the binary and ternary systems was the most sensitive to the changes in the tertiary structure of hTf upon addition of ROP and ASA. The structure of the C-lobe of hTf imposed extra rigidity and less flexibility as opposed to the N-lobe [8,9,31], for which reason the negative band at 319 nm was related to the conformational changes of the N-lobe. Hence, the modifications of the tertiary structure of the C-lobe concerned the negative band at 334 nm. This result demonstrated the partially unfolded states of hTf. 

The H parameter (H = h/h′) depended on the dielectric constant of the solvent and was used as an empirical measure of the effect of the relative hydrophobicity of solvent in the second derivative of the spectra. Here, h is the difference in intensities between the minimum, around 320–350 nm, and the shoulder, at 370 nm, and h′ represents the difference in intensities between the minimum, around 320–350 nm, and the maximum, at 400 nm. H is the only empirical parameter and is related to changes in the degree of polarity in the environment of all the Trp residues in hTf [32].

Figure 3E,F present a plot of the H values versus the concentrations of ROP and ASA for the binary and ternary systems, and as can be seen, a decrease in H values upon addition of ROP and ASA indicated a red shift. The polarity around the Trp residues was increased and the hydrophobicity was decreased, causing a further exposure of Trp residues from the core of hTf to the polar solvent. Figure 3E shows a higher slope for the binding of ROP to hTf, probably due to the conformational changes of the Trp residues and the further exposure to the polar solvent. As can be seen in Figure 3F, the hTf-ASA and (hTf-ROP)-ASA spectra overlapped, revealing that ROP did not affect or only weakly participated in the changes of the tertiary structure of hTf, in the presence of ASA.

### 2.4. Synchronous Fluorescence

The synchronous fluorescence spectroscopy technique was introduced by Lioyd in 1971. The method provides information about the molecular environment in the vicinity of chromophore molecules with several advantages, such as sensitivity, spectral simplification, spectral bandwidth reduction and the avoidance of several perturbing effects. According to the theory of Miller, when the D-value (Δλ) between excitation and emission wavelengths becomes stabilized at 15 nm or 60 nm, the synchronous fluorescence gives the characteristic information of Trp or Tyr residues [33,34]. Thus, the synchronous fluorescence applied to the synchronous luminescence is: 


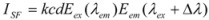
(3)

where I_SF_ is the relative intensity of synchronous fluorescence; E_ex_ is the excitation function at the given excitation wavelength; E_em_ is the normal emission function at the corresponding emission wavelength; c is the analytical concentration; d is the thickness of the sample cell; and k is the characteristic constant comprising the instrumental geometry factor and related parameters. Due to the relationship of the synchronous fluorescence intensity (I_SF_) and the concentration of hTf, I_SF_ should be in direct proportion to the concentration of hTf [35].

The synchronous fluorescence of hTf at Δλ = 15 nm and 60 nm are characteristic of Tyr and Trp residues, respectively. It is considered that the λ_max_ of the Trp residues are relative to the polarity of the micro-environment. As reported, the λ_max_ at 330–332 nm indicates that Trp residues are located in the nonpolar region. In other words, they are buried in a hydrophobic cavity in hTf. The λ_max_ at 350–352 nm shows that Trp residues are exposed to polar solvent, hence, the hydrophobic cavity in hTf is disagglomerated and the structure of hTf is looser [36]. 

**Figure 4:**
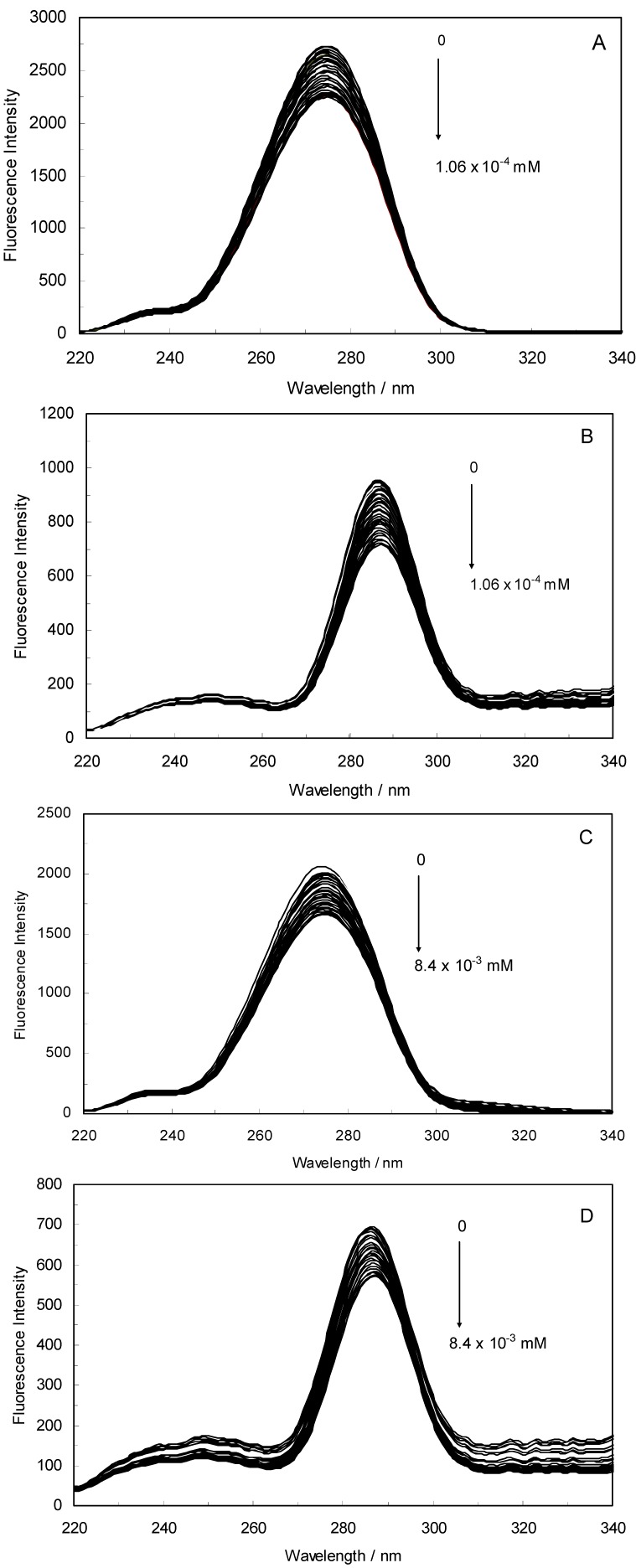
(**A**) Synchronous fluorescence spectra of the hTf-ROP system. [hTf] = 3.8 × 10^−3^ mM and [ROP] was increased from 0 to 1.06 × 10^−4^mM, ∆λ = 60 nm; (**B**) Synchronous fluorescence spectra of the hTf-ROP system. [hTf] = 3.8 × 10^−3^mM and [ROP] was increased from 0 to 1.06 × 10^−4^mM, ∆λ = 15 nm; (**C**) Synchronous fluorescence spectra of the hTf-ASA system. [hTf] = 3.8 × 10^−3^ mM and [ASA] was increased from 0 to 8.4 × 10^−3^ mM, ∆λ = 60 nm; (**D**) Synchronous fluorescence spectra of the hTf-ASA system. [hTf] = 3.8 × 10^−3^mM and [ASA] was increased from 0 to 8.4 × 10^−3^mM, ∆λ = 15 nm.

For the first time, ROP and ASA were used to determine the hTf with a synchronous fluorescence technique. Figure 4 shows the Trp and Tyr residues of the fluorescence spectra of hTf at various concentrations of ROP and ASA for binary systems, respectively. It is apparent from Figure 4A,B that the addition of ROP and ASA to hTf (binary and ternary systems) led to a dramatic decrease in the intensity of fluorescence emission maximum of the Trp and Tyr residues. As seen in Figure 4A,B, the maximum emission wavelength of the Trp and Tyr residues do not have a significant shift. As displayed in Figure 4C,D for hTf-ASA complex and ternary systems (data not shown), the emission maximum of Trp and Tyr residues were red-shifted, which demonstrated that the polarity of the fluorophores increased, causing the hydrophobic cavities to be moved to a more hydrophilic environment.

**Figure 5:**
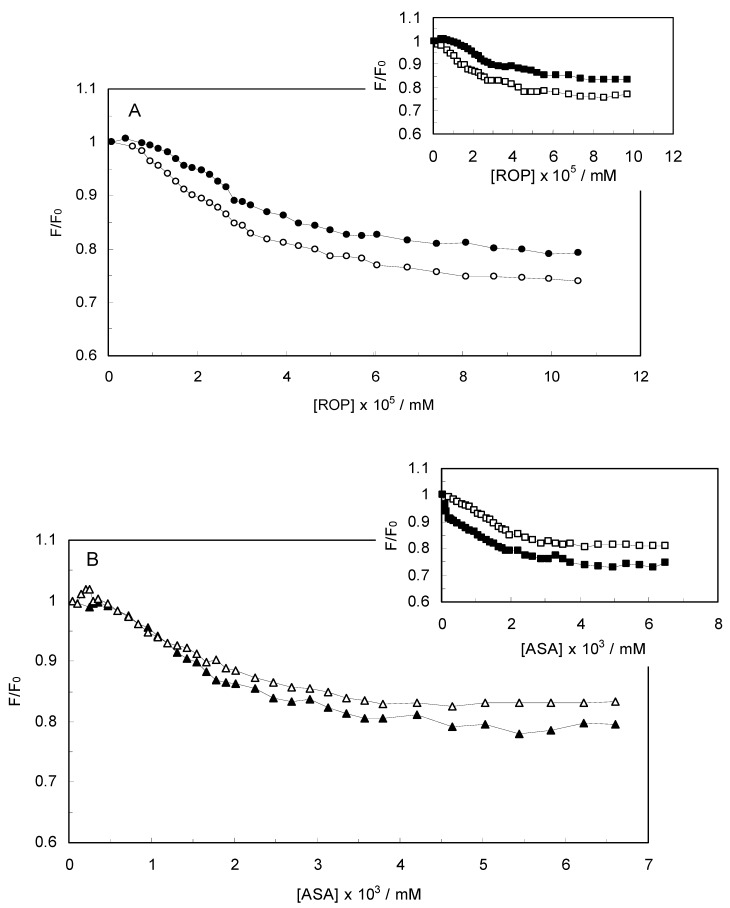
(**A**) Synchronous fluorescence spectra of the quenching of hTf by ROP. (○) ∆λ = 15 nm and (●) ∆λ = 60 nm. **Inset (A)** Synchronous fluorescence spectra of the quenching of hTf by ROP in the presence of ASA. (□) ∆λ = 15 nm and (■) ∆λ = 60 nm; (**B**) Synchronous fluorescence spectra of the quenching of hTf by ASA. (∆) ∆λ = 15 nm and (▲) ∆λ = 60 nm. **Inset (B)** Synchronous fluorescence spectra of (hTf-ROP)-ASA system. (□) ∆λ = 15 nm and (■) ∆λ = 60 nm.

Figure 5A displayed a higher slope when Δλ was 15 nm, indicating a significant contribution of Tyr residues of hTf in the absence and presence of ASA, and ROP closer to Tyr residues as compared to Trp residues. The binding of ROP with hTf when Δλ was 60 nm, revealing a contribution of Trp residues in the fluorescence of hTf (without the ASA), and ROP closer to the Trp residues. The synchronous fluorescence quenching of hTf by ROP, in the presence of ASA, differed only slightly when Δλ was 15 nm (data not shown). Consequently, this suggested that the ROP and ASA approaching the Tyr residues were equal. Figure 5B demonstrates that the slope was similar for the hTf-ASA system whether Δλ was 15 nm or 60 nm, indicating that the opportunity of ASA to approach the Tyr and Trp residues was the same. Hence, it could be concluded that ASA bound to the central cavity of hTf thus forming an hTf-ASA complex [37].

It is also shown, in the inset of Figure 5B that the slope was higher when Δλ was 60 nm, demonstrating a significant contribution of Trp residues of hTf in the presence of ROP, and the fact that ASA was closer to the Trp residues as compared to the Tyr residues. Indeed, the structure of the micro-environment of the Trp residues was altered by ASA in the presence of ROP. The higher slope for (hTf-ROP)-ASA when Δλ was 60 nm, which was the result of a main contribution of Trp residues of hTf in the presence of ROP, bringing ASA closer to the Trp residues (data not shown). One can see that the slope was similar when Δλ was 15 nm, revealing that ASA and ROP in (hTf-ROP)-ASA approached the Tyr residues (data not shown).

### 2.5. Thermodynamic Parameters and Nature of the Binding Forces

The interaction forces a small molecule and macromolecule commonly include hydrophobic force, electrostatic interactions, van der Waals interactions and hydrogen bond, *etc*. The thermodynamic parameters dependent on temperature are calculated according to the van’t Hoff equation in order to elucidate the interaction forces between hTf, ROP and ASA and are listed in Table 1:


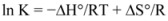
(4)

Thus the enthalpy change (ΔH°) and entropy (ΔS°) for the binding reaction can be determined from a plot of ln K versus 1/T (spectra are not shown). Subsequently the free energy change (ΔG°) is calculated by the following equation: 


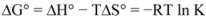
(5)

where K is the binding constant at the corresponding temperature and R is the gas constant [28,29,30,38]. The negative sign for ΔG° means that the binding process was spontaneous. The negative values of entropy indicated that the binding was mainly entropy-driven and the enthalpy values were unfavorable for binary and ternary systems. Ross and Subramanian have characterized the sign and magnitude of the thermodynamic parameter associated with various individual kinds of interaction that may take place in protein association process, which can be easily concluded as: (a) ΔH° > 0 and ΔS° > 0, hydrophobic force; (b) ΔH° < 0 and ΔS° < 0, van der Waals force and hydrogen bond; (c) ΔH° < 0 ΔS° > 0, electrostatic interactions [39]. Thus, from the thermodynamic characteristics summarized above, the negative ΔH° and ΔS° values demonstrate that van der Waals force and hydrogen bond played major roles in the binding of ROP and ASA to hTf and contributed to the stability of the complexes. 

### 2.6. Circular Dichroism (CD) Analysis

Circular dichroism is an extremely convenient technique for detecting and monitoring the extent of conformational changes that may be associated with the activity or regulation of a protein. To further verify the possible influence of ROP and ASA (binary and ternary systems) on the secondary structure of hTf, far-UV CD studies (free and within the mixture) were performed with ROP and ASA. 

**Figure 6:**
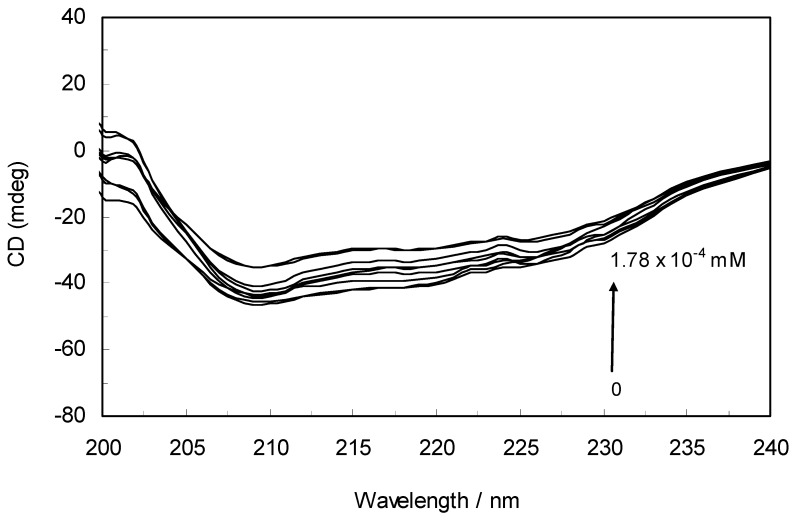
Far-UV CD spectra of hTf in the absence and presence of ROP. pH 7.4 and T = 298 K. [hTf] = 3.8 × 10^−3^ mM. The concentrations of ROP ranged from 0 to 1.78 × 10^−4^ mM.

The CD spectra of hTf in the absence and presence of ROP are displayed in Figure 6. It was apparent that these spectra exhibited two negative bands in the UV region at 208 nm and 222 nm, characteristic of an α-helical structure of hTf. A reasonable explanation is that the negative peaks at 208 nm and 222 nm both resulted from n → π^*^ transfer for the peptide bond of the α-helix. In addition, the band intensities of hTf at 208 nm and 222 nm decreased with the negative cotton effect through the binding of ROP without causing conspicuous shifts of the peaks, clearly indicating that ROP induced a slight decrease in the α-helical structure content of hTf. From the above results, it was evident that the effect of ROP on hTf caused secondary structural changes to the hTf with loss of the helical stability [40]. 

The CD results are expressed as MRE (Mean Residue Ellipticity) in deg cm^2^ dmol^−1^, which is defined as:


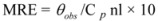
(6)

where *θ_obs_* is the CD in milli-degree; C*_p_* is the mole fraction; n is the number of amino acid residues (679); and *l* is the path length of the cell. The α-helical content could be calculated from the MRE values at 208 nm using the following equation: 


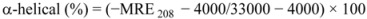
(7)

where MRE_208_ is the observed MRE value at 208 nm; 4,000 is the MRE of the form and random coil conformation cross at 208 nm; and 33,000 is the MRE value of an α-helix at 208 nm.

Table 3 demonstrate the effect of ROP and ASA (in binary and ternary systems) on the relative amounts of α-helical, β-sheet, turn and unordered coil fractions in hTf. Since there occurred a decrease of the content of α-helix whereas there was an increase in β-sheet, turn and unordered coil contents in the hTf-ROP complex, the fraction of secondary structure was closely related to the biological activity of hTf. A decrease in α-helical structure from 37.4% to 33.7% reveals a loss of the biological activity of hTf with the highest concentration of ROP. Hence, hTf adopted a more incompact conformation state [34,35,41,42]. These results indicated an increase of the α-helix fraction and a decrease of the contents of β-sheet, turn and unordered coil in hTf-ASA in the absence and presence of ROP and (hTf-ROP)-ASA complexes. The increase of the secondary helical structure content demonstrated that ASA induced this structure in hTf.

**Table 3:** Fractions of the secondary structure and binding distance (r) values of hTf with ROP and ASA complexes as binary and ternary systems, at pH = 7.4 and T = 298 K.

System	α-helix %	β-sheet %	Turn %	Unordered %	r/nm
hTf	37.4	28.3	15.4	18.9	-----
hTf-ROP	33.7	29.4	16.5	20.4	1.92
(hTf-ASA)-ROP	37.6	28.2	15.2	19.0	2.17
hTf-ASA	40.2	27.4	15.1	17.3	2.13
(hTf-ROP)-ASA	39.5	27.7	15.1	17.7	2.22

### 2.7. Characteristics of the Resonance Light Scattering Spectra

**Figure 7:**
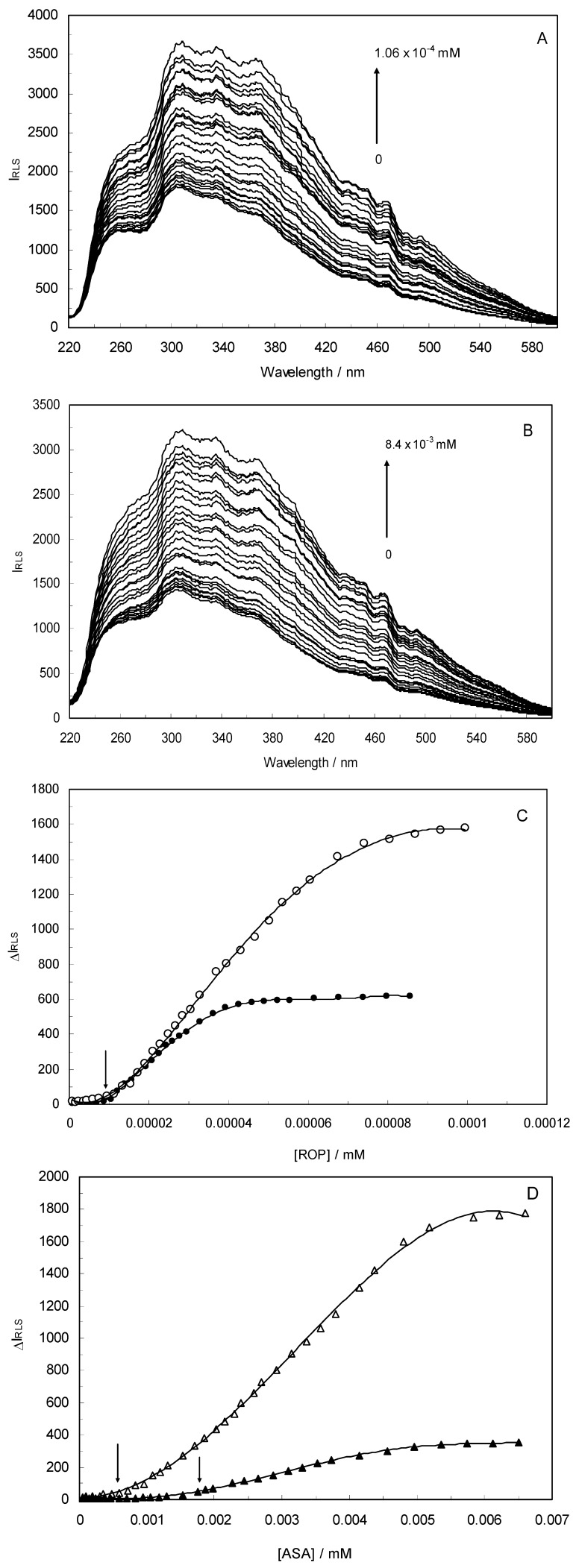
(**A**) The RLS spectra of hTf at varying concentrations of ROP. [hTf] = 3.8 × 10^−3^ mM; (**B**) The RLS spectra of hTf at different concentrations of ASA. [hTf] = 3.8 × 10^−3^ mM, pH 7.4; (**C**) A plot of the ∆I_RLS _intensity of hTf at various concentrations of ROP in the absence and presence of ASA. [hTf] = 3.8 × 10^−3^ mM, and [ASA] = 4.62 × 10^−3^ mM for the ternary system. (○) binary system, (●) ternary system; (**D**) A plot of the ∆I_RLS _intensity of hTf at several concentrations of ASA in the absence and presence of ROP. [hTf] = 3.8 × 10^−3^ mM, and [ROP] = 1.33 × 10^−5^ mM for the ternary system. (∆) binary system, (▲) ternary system. All experiments were performed under identical conditions, T = 298 K, pH = 7.4.

Light-scattering has been widely applied to study the aggregation, size, shape and distribution of particles in solution. When the excitation wavelength is close to the absorption bands, greatly enhanced Rayleigh light-scattering signals can be expected, known as resonance light scattering (RLS). The resonance light scattering spectra of ROP and ASA with hTf (in the binary and ternary systems) became enhanced upon increasing the concentration of drugs (see Figure 7A,B). Under the experimental conditions, the RLS intensity of hTf was high and the coexistence of hTf and a certain concentration of ROP and ASA solutions led to a raise in RLS intensity. The results pointed at the conclusion that ROP and ASA induced aggregation of the hTf, giving rise to an enhancement of the RLS intensity. This followed the formula of RLS:


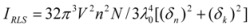
(8)

where n is the refractive index of the medium; N is the molarity of the solution; λ_0_ is the wavelength of incident and scattered light; V^2^ is the square of the molecular volume; and δ_n_ and δ_k_ are the fluctuations in the real and imaginary components of the refractive index of the particle, respectively [43,44]. When other factors are constant, I_RLS_ is related to the size of the formed particle and is directly proportional to the square of the molecular volume (*i.e.*, V^2^). Apparently, with the increase of the molecular volume, I_RLS_ was enhanced. The values of I_RLS _were plotted against varying concentrations of ROP and ASA (in the binary and ternary systems) (see Figure 7) and it was found that the enhancement of the RLS intensity differed as a function of the concentrations of ROP and ASA. When the ROP and ASA concentrations were too low, the RLS intensity of the binary and ternary systems hardly changed. However, with increasing ROP and ASA concentrations, the RLS intensity of these systems gradually increased, and precipitation was even observed to occur in the solutions containing high concentrations of drugs. 

The relationship between the RLS intensity and these drug concentrations was nonlinear. The vertical arrow shows the critical induced aggregation concentrations (C_CIAC_) of ROP and ASA inducing drug aggregation on hTf [45,46].

Under identical experimental conditions, smaller C_CIAC_ values for the ternary systems signified a lower concentration of ROP and ASA for the induction of hTf aggregation. The C_CIAC_ values of the hTf-ROP and (hTf-ASA)-ROP complexes were 9.49 × 10^−6^ mM and 1.04 × 10^−5^ mM, respectively (see Figure 7C,D), and the values for the hTf-ASA and (hTf-ROP)-ASA complexes were 5.92 × 10^−4^ mM and 1.75 × 10^−3^ mM, respectively. The phenomenon indicated that ROP and ASA could precipitate on the hTf in binary and ternary systems.

### 2.8. Binding Distance and Energy Transfer for Binary and Ternary Systems

**Figure 8:**
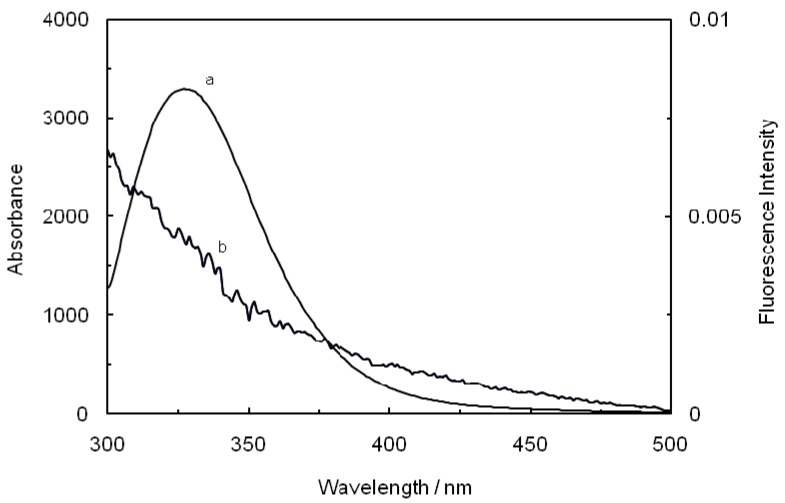
Spectral overlap of the fluorescence spectra of hTf in the presence of ASA (a) with the absorption spectra of ROP (b) at pH 7.4 and T = 298 K. [hTf] = 3.8 × 10^−3^ mM, [ASA] = 4.6 × 10^−3^ mM and [ROP] = 7.27 × 10^−5^ mM.

In order to determine the precise location of ROP and ASA for binary and ternary systems in hTf, the efficiency of energy transfer were studied according to the forster resonance energy transfer theory (FRET), if the emitted fluorescence from a donor could be absorbed by an acceptor, energy may transfer from the donor to the acceptor. Energy transfer will happen under the following conditions: (a) the donor can produce fluorescence light; (b) the absorption spectrum of the receptor overlaps enough with the donor’s fluorescence emission spectrum; (c) the distance between the donor and the acceptor is less than 7 nm. The fluorescence quenching of hTf after binding with ROP and ASA indicated that the transfer of energy have occurred [47]. The following equation can be used to calculate the efficiency (E) of energy transfer between the donor and acceptor:


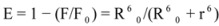
(9)

where F and F_0_ are the fluorescence intensities of the biomolecule in the presence and absence of a quencher, r is the donor- acceptor distance and R_0_ is the critical energy transfer distance, at which 50% of the excitation energy is transferred to the acceptor. It is given by the following equation:


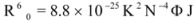
(10)

where K^2^ is the orientation factor of the dipole of the donor and acceptor, N is the refractive index of the medium, Φ is the fluorescence quantum yield of the donor in the absence of the acceptor and J expresses the degree of spectral overlap between the donor emission spectrum and the acceptor absorption spectrum. The value of J is given by:


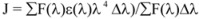
(11)

where F(λ) is the fluorescence intensity of the fluorescent donor at wavelength λ, and ε(λ) is the molar absorption coefficient of the acceptor at wavelength λ. From the above relationships, J and E can be easily obtained, from which follows that R_0_ and r can be further calculated [48]. As seen in Figure 8, there were a considerable overlap between the absorption spectrum of ROP with the fluorescence emission spectrum of hTf in the presence of ASA, which formed the basic of FRET. The average distance r < 7 nm and 0.5 R_0_< r < 1.5 R_0_ indicated that the energy transfer for binary and ternary systems occurred with a high probability.

The parameters regarding the FRET are presented in Table 3. This results suggested that the bound ROP and ASA had a different distance to the tryptophan residues for binary and ternery complexes. The reasons for this, was that the binding sites for ROP and ASA in hTf, depending the chemical structure, molecule’s size and electro negativity of drugs [49]. Moreover, The r values decreased with increasing values of the binding constant and binding affinity for binary and ternary systems.

### 2.9. Time-Resolved Fluorescence

Fluorescence lifetime decay measurements supply one of the best parameters that help us to distinguish between static and dynamic processes. In order to further substantiate the quenching mechanism of ROP and ASA to hTf, fluorescence lifetime of hTf were ascertained in the absence and presence of ROP and ASA as binary and ternary systems. Average fluorescence lifetime (τ) for biexponential iterative fittings was calculated from the decay times and the relative amplitude (α) using the following equation:


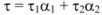
(12)

Time-resolved fluorescence decay of hTf in the absence and presence of ROP and ASA are summarized in Table 4 (the concentrations of hTf, ROP and ASA for binary systems are 3.8 × 10^−3^ mM, 8 × 10^−4^ mM and 5 × 10^−2^ mM, respectively, although for ternary systems the concentrations of ROP and ASA are 7.27 × 10^−5^ mM and 4.6 × 10^−3^ mM, respectively). The average fluorescence lifetime reduces from 4.20 to 3.59 ns, without and with the ROP and ASA for binary and ternary systems, attesting that the fluorescence quenching is essentially static mechanism. Hence, both steady-state and time-resolved measurements hint to the occurrence of static type fluorescence quenching caused by specific interaction, mainly by ground-state complex formations [50,51,52].

**Table 4:** Lifetime of hTf in the presence of ROP and ASA as binary and ternary systems, (α: the relative amplitude and χ^2^: the error in the calculated fluorescence) at pH 7.4, T = 298 K and λ*_ex_* = 295 nm.

System	τ_1_/ns	α_1_	τ_2_/ns	α_2_	τ/ns	χ^2^
hTf	1.957	0.5947	6.125	0.4972	4.209	0.9519
hTf-ROP	1.938	0.7634	6.033	0.3719	3.723	0.9611
(hTf-ASA) ROP	1.894	0.7954	5.772	0.3627	3.599	0.9520
hTf-ASA	1.942	0.7724	6.086	0.3771	3.795	0.9519
(hTf-ROP) ASA	1.907	0.7517	5.921	0.3661	3.601	0.9521

### 2.10. Three-Dimensional Fluorescence Spectra

Three-dimensional fluorescence spectroscopy is a vigorous method for providing conformational and structural information of proteins. The outstanding advantage of three-dimensional fluorescence spectra is that information regarding the fluorescence characteristics can be entirely acquired by simultaneously changing the excitation and emission wavelengths. The maximum emission wavelength and the fluorescence intensity of the residues have a close relation to the polarity of their micro-environment [53]. 

**Figure 9:**
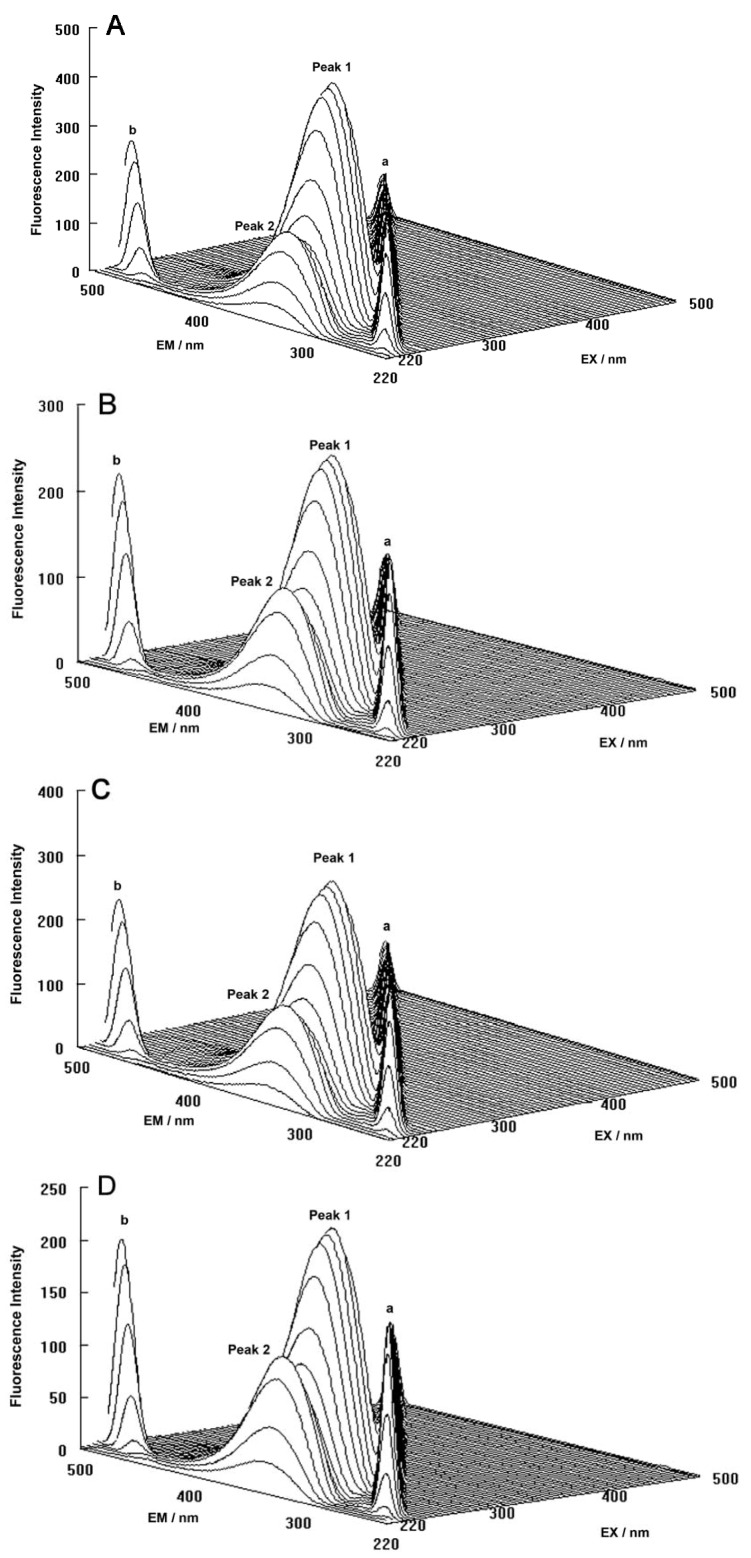
(**A**) Three-dimensional fluorescence spectra of hTf; (**B**) Three-dimensional fluorescence spectra of the hTf-ROP system; (**C**) Three-dimensional fluorescence spectra of the hTf-ASA system; (**D**) Three-dimensional fluorescence spectra of the hTf-ROP-ASA system. All experiments were performed under identical conditions, [hTf] = 3.8 × 10^−3^ mM, [ROP] = 7.27 × 10^−5^ mM, [ASA] = 4.54 × 10^−3^ mM, pH = 7.4 and T = 298 K.

The three-dimensional fluorescence spectra and the contour maps for both the binary and ternary systems are shown in Figure 9A–D. As can be seen, peak a is the Rayleigh scattering peak (λ_ex_ = λ_em_) and peak b is the second-ordered scattering peak (λ_em_ = 2λ_ex_). The fluorescence intensity of peak a and peak b increased with the addition of ROP and ASA (in the binary and ternary systems). The possible reason for this was that complexes came into being after addition of the drugs, increasing the diameter of the hTf which in turn resulted in enhanced scattering spectra.

At the same time, there were two “humps” in the three-dimensional spectra for both binary and ternary complexes marked peaks 1 and 2. Peak 1 revealed the spectral behavior of the Trp and Tyr residues. The reason for this was that when the hTf was excited at 280 nm, it mainly demonstrated the intrinsic fluorescence of Trp and Tyr residues, and the fluorescence of Phe residues could be neglected. The fluorescence spectral behavior of the polypeptide backbone structure of hTf was also reflected by peak 2, which was caused by the transition of π → π^*^ of hTf’s characteristic polypeptide backbone structure C=O. The fluorescence intensity of peak 2 decreased after the addition of ROP and ASA, signifying that the peptide strands structure of hTf had been modified [54,55].

The decrease of the intensity of peaks 1 and 2 in combination with the results from the synchronous fluorescence spectroscopy displayed that the binding of ROP and ASA to hTf induced the slight unfolding of the polypeptides of hTf, which led to a conformational change of hTf that increased the exposure of some hydrophobic regions that were previously buried. All these phenomena obtained from analyzing the two peaks indicated that the binding of ROP and ASA to hTf and the formation of binary and ternary complexes induced certain changes in the micro-environment and conformation of hTf. 

### 2.11. Zeta-Potential Measurements

By measuring the zeta-potential, it is possible to probe a characteristic colloidal property in a complex mixture of particles, making it a useful technique to explain their behavior. The zeta-potential is generated when a liquid is forced to flow directly through a small gap, formed by two sample surfaces, under pressure. The charge carrier bound in the double layer will thus be removed, after which the potential can be measured between two electrodes. Consequently, the biomaterial’s zeta-potential demonstrates the electric surface properties. A surface charge in protein particles is due to the partial ionization of various amino acid residues. The effective charge on a protein particle is affected by pH, ionic strength and the accumulation of ligands or surfactant at the interface [56,57]. The zeta potential can be calculated from the electrophoretic mobility’s, *µ_E_*, using the Henry equation [58]:


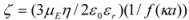
(13)

where *ε*_0_ is the permittivity of vacuum; *ε_r_* and *η* are the relative permittivity and viscosity of water, respectively; α is the particle radius; and κ is the Debye length. The function *f(κα)* depends on the particle shape and was determined for the systems by: 


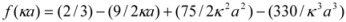
(14)

This expression is valid for *κα* > 1. The interaction between the adsorbed molecules may be either attractive or repulsive, depending on the kind and magnitude of electric charge of the residues. These complicated processes involving protein adsorption are also reflected in the zeta-potential changes.

**Figure 10:**
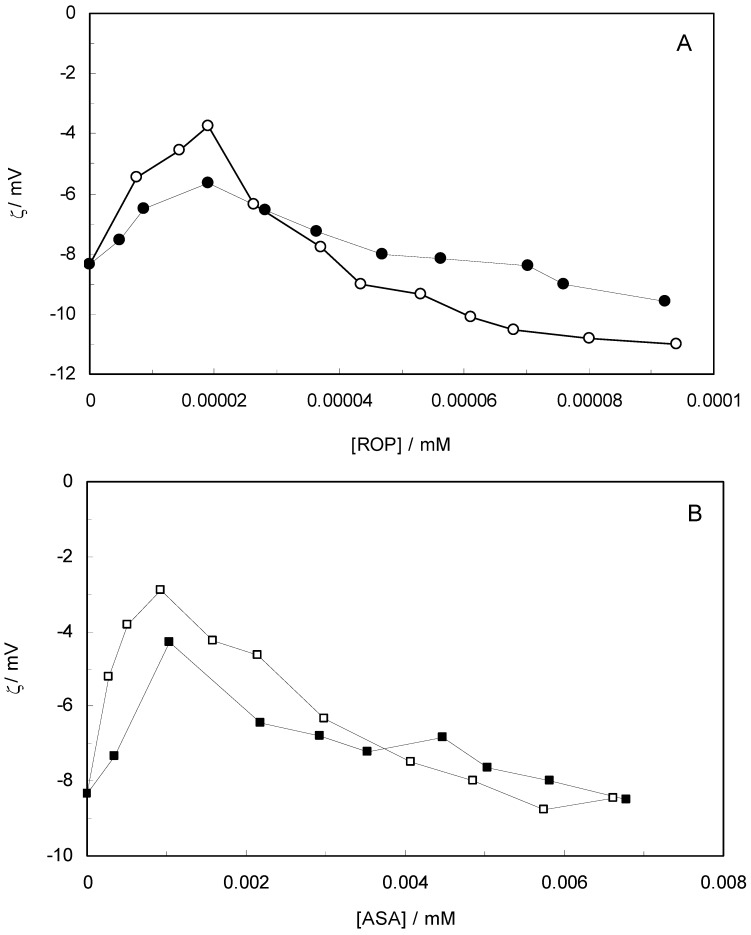
(**A**) Zeta-potential spectra at varying concentrations of ROP in the absence and presence of ASA. [hTf] = 3.8 × 10^−3^ mM, [ASA] = 4.62 × 10^−3^ mM, for the ternary system; (**B**) Zeta-potential spectra at various concentrations of ASA in the absence and presence of ROP. [hTf] = 3.8 × 10^−3^ mM, [ROP] = 1.33 × 10^−5^ mM, for the ternary system. pH 7.4 and T = 298 K.

Figure 10 shows the effect of ROP and ASA concentrations on the zeta-potential of hTf (in the binary and ternary systems). At first, the adsorption of ROP and ASA on the hTf surface increased with an increasing zeta-potential after which an abrupt change occurred, decreasing the values of the zeta-potential. Higher zeta-potential values confirmed that the electrostatic forces were the primary interaction of ROP and ASA with hTf in the present study. The negative values of the zeta-potential conformed with hydrophobic interactions between ROP, ASA and hTf. Hence, the stability of the binary and ternary systems decreased when decreasing the zeta-potential values. The raise in surface charge on the colloidal particles augmented the magnitude of inter-particle electrostatic repulsion, which tended to disrupt existing protein aggregates and discourage further aggregate formation [59]. Consequently, the ROP and ASA molecules were able to bind to hTf through a combination of electrostatic and hydrophobic interactions, forming micelle-like clusters.

### 2.12. Molecular Modeling

**Figure 11:**
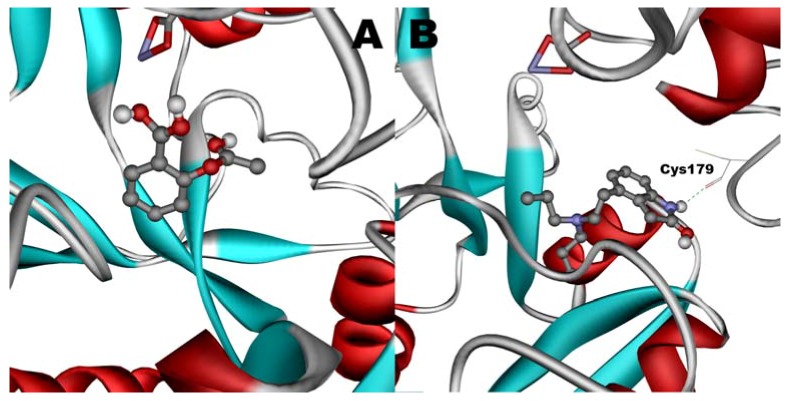
Docking of ASA (**A**) and ROP (**B**) onto hTf. ASA and ROP are shown as balls and sticks. FeCO3, if visible, is shown as sticks. Hydrogen bonds are shown as green dotted lines.

**Figure 12:**
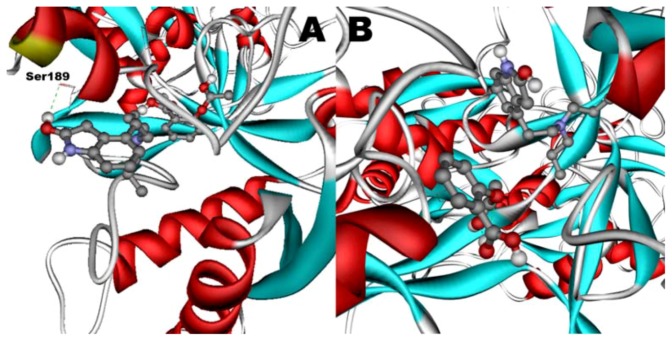
Docking of ASA (**A**) as a third ligand onto the ROP-hTf complex. ROP (**B**) docked onto the ASA-hTf complex. ASA and ROP are shown as balls and sticks. FeCO3, if visible, is shown as sticks. Hydrogen bonds are shown as green dotted lines.

Any of the ligands, when docked in hTf, becomes attached to the N-lobe of the hTf and shows the most affinity to approximately the same active site. The best docking results for binary and ternary systems are shown in Figure 11 and Figure 12, respectively.

When docked alone, ROP formed a hydrogen bond to Cys179 and properly fits the binding pocket (Figure 11B). However, when introduced to the hTf-ASA complex, it could not attach to pocket in the same orientation; and created instead a hydrogen bond to Ser189 (Figure 12B). The inhibition constant declined to about one fourth of the previous value due to poor orientation and improper van der Waals interactions. ASA, although unable to form hydrogen bonds, had a good placement in terms of electrostatic energy and yielded a decent inhibitory constant (Figure 11A). The presence of ROP in the complex forced ASA to dock in a different pocket and stabilize by formation of two intramolecular hydrogen bonds [60,61,62]. It is predicted that presence of ROP when docking ASA shouldn’t have a major effect on affinity (Figure 12A). The molecular dynamic results confirmed the obtained experimental data that mentioned in Table 2.

## 3. Experimental

### 3.1. Materials and Solutions

All reagents were of analytical grade, purchased from the Sigma-Aldrich Corporation, (St. Louis, Mo, USA) and used without further purification. The hTf solution (3.8 × 10^−3^ mM) was prepared at room temperature as a dissolution in a 50-mM potassium phosphate buffer solution with pH = 7.4. The ROP solution (8 × 10^−4^ mM) and an ASA solution (5 × 10^−2^ mM) in potassium phosphate buffer were also prepared. Water was purified with a Milli-Q purification system (Millipore USA) to a specific resistance: 16.4 MΩcm^−1^. All solutions were stored in a refrigerator at 4 °C in the dark.

### 3.2. Apparatuses

#### 3.2.1. Fluorescence Spectroscopy

Fluorescence measurements were obtained using a Hitachi F-2500 spectrofluorometer equipped with a Xenon lamp. The widths of the excitation and emission slits were set to 5.0 nm, and the response time (auto) and excitation voltage (medium) were kept constant for each data set. The excitation wavelengths were set at 280 nm and 295 nm, and the emission wavelength was recorded between 300 nm and 500 nm. The operation software automatically corrected the spectral scan for photomultiplier characteristics. Moreover, the fluorescence intensities were corrected for inner filter and dilution effects before analysis of the binding and quenching data. An appropriate buffer was taken as a blank and subtracted from the experimental spectra to correct for the background of fluorescence. One-centimeter quartz cells were used throughout the experiments and all the measurements were performed at different temperature. The average of five scans was subjected to smoothing using a 10-point smoothing average. Finally, the second derivative of the smoothed spectrum was obtained with the same software. A smoothing step of the normalized data was required to reduce the noise in the second derivative. The criteria for smoothing was that the overall shape and intensity of the raw emission scan was not affected by the smoothing and, at the same time, that the overall shape of the bands in the second derivative was preserved and the excess noise removed. 

#### 3.2.2. Resonance Light Scattering

Resonance light scattering (RLS) spectra were recorded by scanning both the excitation and emission monochromators of a common spectrofluorometer with Δλ = 0 nm. RLS can be developed and has proven to be able to investigate the aggregation of small molecules as well as the long-range assembly of drugs on biological templates. All RLS spectra were obtained by simultaneously scanning the excitation and emission monochromators from 220 nm to 600 nm with slit widths of 5.0 nm for the excitation and emission. 

#### 3.2.3. Synchronous Fluorescence Spectroscopy

Synchronous fluorescence spectroscopy was carried out by simultaneously scanning the excitation and emission monochromators. The spectra only showed the Tyr and Trp residues of hTf, when the wavelength interval (Δλ) was 15 nm and 60 nm, respectively.

#### 3.2.4. Time-Resolved Fluorescence Measurements

Time-resolved fluorescence spectra were executed in a time correlated single photon counting system from ARCUS Fluorometer (LKB, Turku, Finland) with excitation wavelength at 295 nm. The data are fitted to biexponential function by an iterative reconvolution approach by the DAS6 decay analysis software utilizing reduced the error in the calculated fluorescence (χ^2^) and weighted residuals as parameters for goodness of fit. 

#### 3.2.5. UV-Vis Spectroscopy

The UV-vis absorption spectra were obtained with a JascoV-630 spectrophotometer. The optical system was based on a split-beam with a grating band width of 5 nm. The light source was a Xenon lamp. The absorption measurements of all samples were carried out using quartz cells with a 1-cm optical path. All experiments were performed at room temperature.

#### 3.2.6. Three-Dimensional Fluorescence Spectroscopy

Three-dimensional spectra were performed on a Jasco FP-6200 spectrofluorimeter, equipped with 1.0-cm quartz cells. The measurement condition were the following: the excitation and emission wavelengths were recorded between 220 nm and 500 nm with an increment of 5 nm, the number of scanning curves was 31 and the other scanning parameters were identical to those of the fluorescence emission spectra.

#### 3.2.7. Circular Dichroism Spectroscopy

Far-UV CD experiments were performed on a Jasco-815 spectropolarimeter equipped with a Jasco 2-syringe titrator. Spectra were recorded with the same protein concentration in a 1-mm path length quartz cuvette. A bandwidth of 1 nm and a response of 2 s were used with a scanning rate of 50 nm min^−1^ to obtain the final spectra as an average of five scan. The instruments were calibrated with ammonium d-10-camphorsulfonic acid. The induced ellipticity, given in degrees, was obtained by the ellipticity of the drug-protein mixture after subtraction of the ellipticity of the drug at the same wavelength. The results are expressed as the mean residue ellipticity [θ], defined as [θ] = 100 × θ_obsd_/(LC), where θ_obsd_ is the observed ellipticity in degrees, C is the concentration in residue mol cm^−3^, and L is the length of the light path in cm. All pH measurements were performed with a Metrohm digital pH-meter (Metrohm, Germany).

#### 3.2.8. Zeta-Potential Measurements

Colloidal particles accumulate charge at their surface, which can be expressed as a surface potential. The surface potential is an important factor for determining the magnitude of charged-based colloidal interactions of a particle, most crucially electrostatic repulsion of other charged particles. The surface charge on a particle perturbs the ionic distribution in the medium surrounding it. First, a layer of tightly bound counter-ions accumulates at the particle surface, *i.e.*, the Stern layer, and beyond this, a region of decaying excess concentration, the diffuse layer, extends a considerable distance (~nm) into the surrounding aqueous media. Measuring the colloidal charge typically involves applying an electrical voltage to the particle and determining the speed of the induced movement. The zeta-potential measurement was performed by using the Zeta-sizer (Nano-ZS) from Malvern Instruments and Dispersion Technology Software (DTS) Zetamaster 5002 by taking the average of five measurements at the stationary level. The cell used a 5 nm × 2 nm rectangular quartz capillary. The temperature of the experiments was 298 ± 0.01 K. All measurements were carried out on hTf, ROP and ASA solutions with concentrations of 3.8 × 10^−3^ mM, 8 × 10^−4^ mM and 5 × 10^−2^ mM, respectively. 

### 3.3. Procedures

hTf, ROP and ASA were dissolved in potassium phosphate buffer, and their concentrations were 3.8 × 10^−3^ mM, 8 × 10^−4^ mM, 5 × 10^−2^ mM, respectively. Aliquots of ROP and ASA were injected into the hTf solution at 5-min intervals to allow for equilibration, and each experiment was repeated three times. The hTf solution was added to a quartz cell to make up 2 mL and the range of (1) a single drug (ROP or ASA) and (2) the two drugs, was gradually titrated manually in the cell using a micro-injector. The fluorescence spectra were then measured (the excitation wavelengths were 280 nm and 295 nm, and the emission wavelength was 300-500 nm). Both the entrance and exit slit widths were 5 nm and the scanning speed was 240 nm min^−1^, rendering it possible to obtain both fluorescence quenching spectra and synchronous fluorescence spectra. The far-UV CD spectra of hTf, ROP and ASA were recorded and spectral scanning curves were obtained under identical conditions. All solutions were freshly prepared for each experiment.

### 3.4. Molecular Modeling

The crystal structure of human transferrin in complex with its receptor was retrieved from PDB (1) (PDB ID: 1SUV(2)). Unnecessary parts were removed using ViewerLite. N-lobe and C-lobe were overplayed in Swiss PDBViewer with respective stand-alone structures from PDB (PDB ID: 1A8Efor N-lobe and for C-lobe 3K0V) to ensure that the conformation of the protein had not changed during the course of attachment. The compounds were energy-minimized by means of AM1 semi empirical force field in the Hyperchem software. The energy-minimized ligands were further processed using an Autodock tools program (3). Partial atomic charges for each atom were added and the rigid root and rotatable bonds for each ligand was calculated automatically. Docking was primarily done using a grid box covering the whole protein to find the active site. A grid map for the entire protein structure was generated with the default 1.000 Å spacing by means of the Autogrid program. The sigmoidal distance-dependent electric permittivity of Mehler and Solmajer was utilized for the calculation of the electrostatic grid maps. The Lamarkian genetic algorithm method was applied for minimization. Default parameters were used and random orientations and torsions were employed for the ligands. After finding the active site, docking was performed with the same parameters in a limited grid box of 0.500 Å spacing. In a final step, the ternary docking was performed after energy minimization of the binary system through a molecular mechanics method with a minimum RMS gradient of 10 kcal/mol or a maximum of 32767 cycles.

## 4. Conclusions

This paper has described an investigation through various spectroscopic techniques, zeta-potential measurements and molecular modeling of the interactions of ROP and ASA with hTf (in binary and ternary systems) at pH 7.4. The quenching of the intrinsic fluorescence and a red shift in the maximum wavelength indicated that an increased polarity of the micro-environment of the fluorophores in hTf was induced by the binding of ROP and ASA as binary and ternary systems. The quenching rate constant, binding constant, number of binding sites and corresponding thermodynamic parameters were calculated according to the relevant fluorescence data. The effect of the displacement of one drug from the complex of the other with hTf has been described on the basis of the comparison of quenching curves and binding constants. From the steady-state and time-resolved fluorescence illustrated that the quenching are static mechanism for binary and ternary complexes. Negative values of thermodynamic parameters namely enthalpy change (ΔH°) and entropy change (ΔS°) indicated that van der Waals force and hydrogen bonds were the dominant intermolecule force in stabilizing the complexes. The results of synchronous fluorescence and three-dimensional fluorescence spectra demonstrated that the structure of the micro-environments of the Trp and Tyr residues was altered by ROP and ASA. The interaction of ROP and ASA with hTf resulted in an enhancement of the RLS intensity, proposing a method for the determination of binary and ternary systems. Therefore it was possible to determine the critical induced aggregation concentration (C_CIAC_) of ROP and ASA inducing the hTf aggregation. The binding distance and the energy transfer efficiency between hTf, ROP and ASA were manifested. The circular dichroism (CD) data revealed that the presence of ROP decreased the α-helical content of hTf, whereas for other complexes, the fraction of α-helix increased. The ROP and ASA molecules bound to the hTf through a combination of electrostatic and hydrophobic attractions. The competition of these drugs in the binding to hTf pointed at precautions being required in a treatment when combinations of drugs are used at the same time. In conclusion, both ROP and ASA formed a binding site in the N-lobe of hTf, thus necessitating the use of monitoring therapy owning to the possible increase of uncontrolled toxic effects. 
